# Bereaved parents’ perspectives on their child’s end-of-life care: connecting a self-report questionnaire and interview data from the nationwide Paediatric End-of-LIfe CAre Needs in Switzerland (PELICAN) study

**DOI:** 10.1186/s12904-022-00957-w

**Published:** 2022-05-04

**Authors:** Karin Zimmermann, Katrin Marfurt-Russenberger, Eva Cignacco, Eva Bergstraesser

**Affiliations:** 1 Department Public Health (DPH), Nursing Science, University of Basel, Bernoullistrasse 28, 4056 Basel, Switzerland; 2 Paediatric Palliative Care and Children’s Research Center CRC, University Children’s Hospital Zurich, Steinwiesstrasse 75, 8032 Zurich, Switzerland; 3 Children’s Hospital of Eastern Switzerland, Claudiusstrasse 6, 9006 St. Gallen, Switzerland; 4 Health Department, Bern University of Applied Sciences, Bern, Switzerland

**Keywords:** Paediatrics, Palliative care, Terminal care, End of life, Parents, Experiences, Mixed methods

## Abstract

**Background:**

Paediatric Palliative Care (PPC) focuses on ensuring the best possible quality of life for the child and his/her family by extending beyond the physical domain into psychosocial and spiritual wellbeing. A deep understanding of what is important to parents is crucial in guiding the further evaluation and improvement of PPC and end-of-life (EOL) care services. Much can be learned from specific positive and negative experiences of bereaved parents with the EOL care of their child. This report builds upon a questionnaire survey as part of the national Paediatric End-of-LIfe CAre Needs in Switzerland (PELICAN) study.

**Methods:**

One part of the PELICAN study was set up to assess and explore the parental perspectives on their child’s EOL care. Interview data were used to explain the extremely positive and negative results of a quantitative survey in an explanatory sequential mixed-methods approach. Data integration occurred at different points: during sampling of the interview participants, when designing the interview guide and during analysis. A narrative approach was applied to combine the qualitative results reported here with the already published quantitative survey results.

**Results:**

Eighteen mothers (60%) and twelve fathers (40%) participated in 20 family interviews. All parents reported having both positive and negative experiences during their child’s illness and EOL, which was characterised by many ups and downs. The families transitioned through phases with a prospect of a cure for some children as well as setbacks and changing health status of the child which influenced prognosis, leading to the challenge of making extremely difficult decisions. Severely negative experiences still haunted and bothered the parents at the time when the interview took place.

**Conclusions:**

A deep understanding of the perspectives and needs of parents going through the devastating event of losing a child is important and a prerequisite to providing compassionate care. This complex care needs to recognise and respond to the suffering not only of the child but of the parents and the whole family. Communication and shared decision-making remain pivotal, as do still improvable elements of care that should build on trustful relationships between families and healthcare professionals.

## Background

Experiencing the end of life (EOL) and death of one’s own child is one of the most devastating events parents may have to live through. During such a shattering crisis, needs-driven, family-centred and compassionate professional care is crucial, as negative experiences during this extremely vulnerable time can haunt parents for many years after the death of their child [1]. Parental needs during their child’s palliative care and/or EOL phase have been investigated extensively [2, 3] and several themes of met and unmet needs emerge consistently: a sincere relationship as well as emotional, spiritual and cultural support; genuine communication; decision making; alleviation of suffering; accessibility, continuity and coordination of care; respite care; bereavement support; support for siblings; and overarching quality of care [2, 3]. Themes such as professional communications skills and coordination of care and respite care are commonly described as unmet [2] and negatively experienced by affected parents [4].

Despite this plethora of parental needs, robust tested instruments to measure parental perspectives on the care provided in the context of paediatric palliative and EOL care are very limited in number [5]. Gill et al.'s scoping review revealed that across the 44 studies included, six articles reported using seven different instruments, only two of which were developed for the field of paediatric palliative care (PPC) [3]. This lack of appropriate systematic inquiry might be a reason why the healthcare system partially fails to acknowledge the various specific needs of dying children and their families [2, 3]. Thus, further efforts should be made to understand the circumstances leading to impaired quality of care. An understanding at a level that goes beyond the contributions of quantitative or qualitative methods separately.

The two instruments developed and tested specifically for paediatric palliative and EOL care show promise for quantitative assessment of the parental perspective [6, 7]. One of them, the Parental PELICAN questionnaire (PaPEQu) was used in the nationwide Paediatric End-of-LIfe CAre Needs in Switzerland (PELICAN) study. The PELICAN study (2012–2015) aimed to provide comprehensive information and understand the current practice of EOL care in paediatric settings in Switzerland. Furthermore, it described and explored parental perspectives of their child’s EOL care and the perspectives of the healthcare professionals involved. The study collected qualitative interview data sequentially for the quantitative questionnaire survey [8]. The PaPEQu specifically assesses the experiences and needs of bereaved parents who have lost a child due to a cardiac, neurological or oncological condition or during the neonatal period, defined as the first four weeks of life. It was developed and validated by the PELICAN study group [6]. The questionnaire is thematically structured along with the six quality domains identified by the Initiative for Paediatric Palliative Care back in 2005 [9] and slightly adapted by Truog et al. [10]. The six domains were as follows: support of the family unit, communication, shared decision-making, relief from pain and other symptoms, continuity of care, and bereavement support. The domain bereavement support encompassed not only the time after the death but also the time around the child’s death. It combines multi-item scales measuring parental experiences along with the six domains as independent latent constructs and single closed questions in dichotomous and multiple-choice formats [6]. Results of this quantitative assessment were published in an earlier issue of this journal [4]. In summary, the domain-specific scale scores showed overall positive parental experiences with their child’s EOL care in a heterogeneous paediatric inpatient and community care setting. Nevertheless, extreme positive values and statistically negative outliers were present raising questions that could not be answered with these quantitative results alone [4]. To gain a deeper understanding of factors explaining especially the negative outliers, we drew on the yet unpublished qualitative data from the PELICAN study to provide a solid basis for the overall interpretation of a deeply distressing parental experience. We specifically aimed (1) to report on qualitative data from the PELICAN study that were concerned with parental experiences about the care at their child’s EOL, and (2) to explain published quantitative questionnaire data by combining them with unpublished qualitative interview data from the PELICAN study.

## Methods

### Setting, design and participants

One part of the nationwide PELICAN study [8] was set up to assess and explore the parental perspective of their child’s EOL care (i.e., in this study, the last 4 weeks of life) incorporating a retrospective survey and interviews. The purpose of this explanatory sequential mixed methods approach was to use interview data to explain initial quantitative results [11]. Mixed methods research is defined as “a research design with philosophical assumptions as well as methods of inquiry.” “Its central premise is that the use of quantitative and qualitative approaches, in combination, provides a better understanding of research problems than either approach alone.” [12, p. 5)] We used mixed methods to provide additional insights into a barely investigated phenomenon, insights that go beyond the contributions of quantitative or qualitative methods separately [13].

Swiss parents who lost a child due to a cardiac, neurological or oncological condition or during the neonatal period in the years 2011 and 2012 were invited to participate in this part of the PELICAN study. Eligible parents were identified by all Swiss children’s hospitals and paediatric units, long-term institutions, and paediatric community care services. Bereaved parents were not invited when their child died within the first 24 h of her/his life. Recruitment took place between July 2013 and March 2014, i.e., parents were bereaved for one to three years. The detailed recruitment strategy along with the results of the survey with the PaPEQu have been reported and published in an earlier issue of this journal [4]. The PELICAN study was approved by Human Research Ethics Committees in all 11 Swiss cantons where recruitment took place (leading committee: KEK ZH Nr. 2012–0537).

Results from the PaPEQu’s experience-related scale scores of each domain sequentially guided the purposeful sampling strategy for the interviews. Parents who showed maximal positive or extremely negative results, representing a statistical outlier in the negative range, i.e. 3^rd^ quartile + 1.5*interquartile range in the PaPEQu survey, and who had already consented at the time of recruitment to possibly participate in an interview, were contacted by phone. The four main diagnostic categories of the PELICAN study (cardiology, neonatology, neurology, and oncology) were additionally used as strata to ensure the same representation as in the survey sample [4]. Applying this stratified sampling strategy and recruitment resulted in a purposeful sample of a total of 30 parents of 20 patients. No families that were contacted refused to participate in the interview.

### Data collection, management and measurements

The survey with the PaPEQu instrument took place between April and June 2014. Since parents consented to study participation before receiving the questionnaire, the response rate was high at 89%, resulting in a complete final PELICAN sample of 200 questionnaires (112 mothers and 88 fathers from 135 families). This quantitative data was then analysed and reported [4].

The response options for the PaPEQu’s 24 experience-related scale items were either a 7-point (0 to 6) with end-point anchors (“never-always”) or a 5-point Likert-type (1 to 5), where respondents indicated the extent to which they agreed with the statement. Lower numbers represented more negative experiences. Detailed results of the initial validation of the PaPEQu are reported elsewhere [6], and a complete list of items is available as an additional file of the earlier publication in this journal [4].

All 20 semi-structured interviews took place in the family’s home between January and May 2015. Where both mother and father participated, the couples wished to be interviewed together. Three experienced and trained interviewers conducted the interviews in their mother tongue corresponding to the parents’ mother tongue, i.e. French, Swiss-German, and Italian. The interviews were initiated with a few instructions followed by a short summary of the aggregated PaPEQu survey results of the whole sample and specific individual results, leading to an open question about the parents’ positive or negative experiences of their child’s EOL care. The interview guide was structured along with the PaPEQu’s six domains, letting those topics guide the interview whenever the need for probing questions arose. Although the PELICAN study focused on the last four weeks of life of the deceased children, parents needed to tell the entire story of their child’s illness and this flow was not restricted by the interviewers. All interviews were audio-taped.

**Fig.1:**
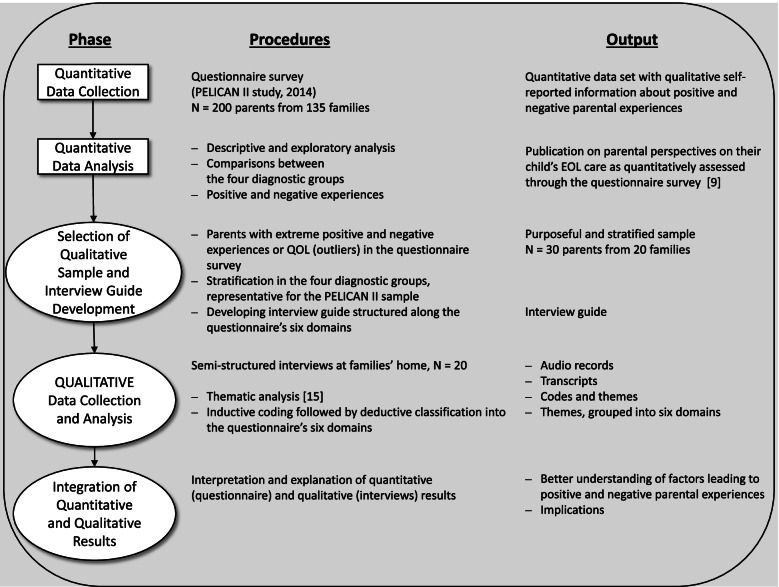
Overview of this study’s explanatory sequential mixed methods approach

Data integration (mixing) occurred at various points: during sample selection, data collection data analysis, and data interpretation [14]. A diagram illustrating how we applied the explanatory sequential mixed method approach in this study is shown in Fig. 1.

### Quantitative and qualitative analysis

Analysis of quantitative and qualitative data was conducted separately in the first step.

#### Quantitative

Quantitative analyses of the complete PELICAN survey sample (*N* = 200) involved a descriptive and exploratory approach applying generalised estimating equations (GEE) and Pearson’s chi-square or Fisher’s exact test for the comparisons between diagnostic groups (cardiology, neonatology, neurology, oncology) [4]. Scale scores were explored descriptively and are presented here as *M (SD)* and *Mdn (range)* due to small diagnostic subsamples and non-normal distributions. IBM® SPSS® Statistics 24 for Mac® (IBM Corp, Armonk, NY, USA) was used for all statistical analyses. All quantitative analyses were performed as part of the initial quantitative reporting [4] and no new analyses were done for this mixing study. In this study, we only report on the quantitative data of the 30 interview participants exclusively.

#### Qualitative

All audio-taped interviews were transcribed verbatim in the language of the record, i.e. French, Swiss-German and Italian. The respective interview language is indicated as French, German, or Italian by the quotes. The median duration of the 20 interviews was 81 min with a range of 37 – 123 min. Qualitative analysis was guided by thematic analysis as described by V Braun and V Clarke [15] and was already started during data collection. Inductive coding was followed by deductive classification into the six domains. All domains were well represented in the interview codes, and a few additional themes emerged repeatedly throughout data collection. Interpretation of qualitative data was discussed within the study group and summarised in an unpublished report. The atlas ti.7 software (ATLAS.ti Inc., Berlin, Germany) was used for data management.

In a second step, we interpreted quantitative and qualitative data using mixed methods to reveal new information that would not be available from the analysis of either data source on its own. A narrative approach was used to interpret and present the integrated quantitative and qualitative data. Findings from the two approaches were amalgamated within the six quality domains, a technique described as weaving by [14].

## Results

Eighteen mothers (60%) and twelve fathers (40%) participated in the 20 family interviews, of whom all 30 parents completed the PaPEQU. Characteristics of this qualitative mixed methods sample are described in Table 1.

**Table 1:** Sample characteristics of parents participating in the interviews

Characteristics	Total *N* = 30 (100%)	Cardiology *n* = 3 (10%)	Neonatology *n* = 14 (47%)	Neurology *n* = 6 (20%)	Oncology *n* = 7(23%)
Age^a^,*M* (*SD)*	40 (7.51)	40 (7.21)	37 (6.27)	37 (3.27)	50 (5.50)
Education, *n* (*%*)					
School levels^b^	1 (3)	0 (0)	0 (0)	0 (0)	1 (14)
Post-school education^c^	13 (44)	1 (33)	7 (50)	2 (33)	3 (43)
Tertiary level^d^	9 (30)	0 (0)	3 (21)	3 (50)	3 (43)
University degree	7 (23)	2 (67)	4 (29)	1 (17)	0 (0)
Family income^e^, *n* (*%*)	*N* = 24	*n* = 2	*n* = 13	*n* = 4	*n* = 5
≤ CHF 100,000.-	12 (50)	0 (0)	8 (62)	1 (25)	3 (60)
> CHF 101,000.-	12 (50)	2 (100)	5 (38)	3 (75)	2 (40)

^a^Age at the time of the survey

^b^Consists of primary and secondary level

^c^Consists of college and vocational education

^d^Consists of degrees from schools of higher education

^e^Annual gross pay, the Swiss average for families with children was CHF 143,000.- in the year 2015[16]

All but two (7%) interview participants were married or living in a partnership at the time when the index child died; 14 participants (47%) had three or more children, the deceased one included, 13 (43%) had two children, and 3 (10%) participants had the deceased child as the only one. All the participating parents’ children died in the hospital, 14 of them (70%), including all neonates and children with heart disease, in an intensive care unit (ICU). The children’s ages differed considerably, depending on the diagnostic group, ranging from 5 days (neonatology) to 10 years (oncology).

### Positive and negative parental experiences

To gain insight and a better understanding of negative parental experiences with their child’s EOL care, interview parents with negative experiences were contrasted with those with positive experiences. The corresponding experience-related scale scores (*M* and *Mdn*) for each of the six quality domains and per diagnostic group are displayed in Table 2, including explanatory quotes of negative experiences for statistical outliers. All parents expressed experiences of uncertainty, unpredictability and vulnerability. Neonates’ family stories commonly began before the birth of their child and many experienced concerns and fears concerning the health of their unborn child, which were not always taken seriously by their physician. The mothers described their exceptional situation of being a mother of a dying child while being a patient themselves at the same time, an aspect that was not revealed by the questionnaire survey.

**Table 2:** Experiences with their child’s EOL care from interview parents (*N* = 30)

Quality domain	Total	Cardiology	Neonatology	Neurology	Oncology	Explanatory quote of a negative experience^a^
(Number of items)	*M (SD) *	*M (SD) *	*M (SD) *	*M (SD) *	*M (SD) *	
	*Mdn*, (*range*)	*Mdn* (*range*)	*Mdn* (*range*)	*Mdn* (*range*)	*Mdn* (*range*)	
Support of the family unit (4)	4.98 (1.13)	4.92 (1.46)	5.16 (1.19)	4.88 (0.75)	4.75 (1.34)	*“I was all alone* (on the obstetrics ward)*. The staff just brought the meals and did not say much. I don’t know, it was as if I was leprous.”* (Mother, Neonatology, G6^b^)
	5.25 (1.25 – 6.00)	5.5 (3.25 – 6.00)	5.25 (1.25 – 6.00)	5.00 (3.50 – 5.50)	5.25 (2.75 – 6.00)	
Communication (6)	4.33 (1.41)	4.33 (0.60)	4.83 (1.12)	3.5 (1.37)	3.97 (2.06)	*“I stayed at the hospital 12 h every day, at my child’s side, and just when I briefly left for a smoke, this physician disregarded my wishes again and spoke to my child about stopping the treatment.”* (Father, Oncology, F7 ^b^)
	5.00 (1.17 – 6.00)	4.12 (3.83 – 5.00)	5.12 (1.83 – 6.00)	3.00 (2.33 – 5.33)	5.08 (1.17 – 5.50)	
Shared decision-making (3)	4.62 (1.53)	3.78 (3.29)	4.23 (1.55)	5.33 (0.36)	5.09 (0.99)	*“We spent days waiting without hearing anything from the physicians, without receiving a decision saying which direction it would go. Not a sign that they even knew what to do. So, we thought in that case we’ll have to decide ourselves.”* (Mother, Cardiology, G2 ^b^)
	5.00 (0.00 – 6.00)	5.33 (0.00 – 6.00)	4.67 (0.33 – 6.00)	5.33 (5.00 – 6.00)	5.33 (3.00 – 6.00)	
Relief of pain and other symptoms (3)	4.90 (1.17)	4.67 (1.53)	4.95 (1.19)	4.28 (1.42)	5.43 (0.57)	*“Our child was screaming in pain. For us (parents) they did not give her/him enough pain killers. Our child cried and cried. They did not assess her/him properly when we arrived (in the hospital), they did not realise the gravity of the situation.”* (Mother, Neurology, F2 ^b^)
	5.00 (1.67 – 6.00)	5.00 (3.00 – 6.00)	5.33 (3.00 – 6.00)	4.83 (1.67 – 5.33)	5.33 (4.67 – 6.00)	
Continuity and coordination of care (4)	4.42 (1.37)	3.94 (2.39)	4.41 (1.53)	3.94 (0.26)	4.97 (0.83)	*“You see, we already had the physiotherapist and ergotherapist as reference persons. When we arrived at the hospital there were all these new people and they knew absolutely nothing about our child and the situation.”* (Mother, Neurology, F2 ^b^)
	4.50 (1.13 – 6.00)	3.94 (2.25 – 5.63)	4.50 (1.13 – 6.00)	3.94 (3.75 – 4.13)	5.25 (3.75 – 5.63)	
Bereavement support (4)	53 (1.29)	3.94 (1.33)	5.62 (0.63)	5.12 (0.78)	4.69 (2.6)	*“We were told we should be glad that our child was able to die as it would have been much more difficult if she/he had collapsed on the way to school with a friend. … You cannot say that death is a good outcome!”* (Mother, Cardiology, G2 ^b^)
	6.00 (0.75 – 6.00)	3.94 (3.00 – 4.88)	6.00 (4.13 – 6.00)	4.87 (4.50 – 6.00)	6.00 (0.75 – 6.00)	

^a^Each quote represents a statistical outlier

^b^Language code (German, French) and interview number

In all interviews, the parents freely told their stories about their premature or sick children. Their experiences were still very present, without signs of a recall bias preventing them from remembering things. The six quality domains that structured the PaPEQu were explicitly identified in all 20 interviews.

#### Support of the family unit

Important aspects of family support mentioned by parents included being allowed to stay with their child 24 h a day and to be supported in maintaining a close relationship with their child through all phases of her/his illness. Being respected in their parental role and expertise for their child by the professionals and the professionals’ interest in their child were essential elements of a trusting relationship.

*“Ms C. and Mr P. (health care professionals, HCPs), they were the ones that stayed with me, the ones we kept in touch with afterwards. It simply stands and falls with the people. I think they are all technically competent, but not all are socially competent. Certain ones were both and those stood out.”* (Father, Neonatology, German interview 5).

The process of building up a trusting relationship has been described as forming a “circle” with HCPs they trusted and was a recurrent theme described by several families.

*“We spent a lot of time in the hospital, could be there 24/7. It is hard to explain, certain people were in our circle. We accepted those HCPs because they were in our circle. Other HCPs who tried to enter, over time, were difficult for us to accept. We did not know them, and they did not know our child. We thought they could not understand us, nor help us. Therefore, we refused their support.”* (Father, Neonatology, French interview 6).

Parents who showed low values on the support scale in the questionnaire survey mainly talked about negative experiences regarding relations with HCPs. Those negative experiences included encounters with professionals who did not respect them as experts for their children and did not meet them as partners. One mother with a median scale score of 1.25 mainly felt not taken seriously by the HCPs and consequently felt left alone.

#### Communication

Good communication was essential for parents and a prerequisite for shared decision-making. Good communication was described as honest, continuous and repetitive, in an adapted language matching the parents’ mother tongue. Importantly, parents did not want to be spared bad news. They appreciated HCPs who were well prepared and took their time to sit down for sharing information.

*“He was such a sweetheart* (physician)*. And the nurse who spoke Italian as well. We had those two *primary* caregivers, and with them we sat at the table every day. They explained what the situation was, how the treatment went and how our child reacted to it.”* (Mother, Neonatology, Italian interview 4).

Expressions of negative experiences were mostly related to non-honest communication or missing information and encounters with HCPs that were not based on equal footing. HCPs needed to address respectfully their child, as a human being and not as a medical case, especially when the child was in a cognitively impaired state.

*“She* (physician) treated* our child as an object. She was interested in how the joints were and the child’s muscle tone. She never reacted to our child as a person.”* (Mother, Neurology, German interview 7).

#### Shared decision-making

All interview parents faced difficult decisions for them to take. They experienced different processes as to how decisions were made. They described them as: shared decision-making between them and the HCPs, physicians putting pressure on them to support the right decision in the best interests of their child and being left alone in the decision process because the physicians sometimes did not know what to do due to the highly complex clinical situation. Parents who experienced good communication in general tended to have experienced processes of shared decision-making.

*“For me, that was a sign from my child, saying ‘I don’t want to go on – let me go’. And then, during that night, I spoke twice with the physician – for a long time. She took time and we talked about the whole situation, and she encouraged me. When I felt that this was right for my child, I could let him go.”* (Mother, Neurology, German interview 3).

The most difficult decisions to take were those involving the withdrawal of life-sustaining measures, decisions which had to be made by all interviewed parents of neonates. Some of the parents of neonates expressed their concerns about feelings of killing their child by deciding to withdraw something sustaining, e.g., ventilation. They all wanted to be part of the decision process but did not want to take this specific decision:

*“I would have preferred for them to decide because for me, I felt too guilty to decide. Even though *we* understood perfectly why to withdraw it, to say that we wanted our child to pass away, that is impossibly hard.”* (Mother, Neonatology, French interview 5).

#### Relief of pain and other symptoms

All interview parents were concerned about the child’s QOL, mostly defined through the child’s suffering. They mentioned the child’s QOL as an important aspect to take into consideration when making decisions. Experiences around pain and its management were described by all parents. Of a variety of symptoms that caused suffering and were listed in the questionnaire survey, the pain was judged by a quarter of the interviewed parents as the most stressful one. Another symptom that was highly stressful to parents was their child’s breathing difficulties. It was described as the dominant stressful burden in the complete sample of the quantitative survey [4].

*“Well, I have this horrible image of my child not getting enough air.”* (Mother, Cardiology, German interview 2).

Especially parents of a child from the neurology group often felt that the HCPs could not help them with managing their child’s symptoms in everyday life. Those families usually took the lead in symptom management and were the ones to initiate adaptations of the care plan – however, without the support of their physicians. Often, they found support in allied HCPs like nurses or physiotherapists, or through exchanges with other affected families. The parents’ fight for appropriate symptom management was closely linked to shortcomings in the continuity and coordination of care, including advance care planning (ACP).

#### Continuity and coordination of care

Continuity and coordination of care were ensured when the HCPs formed a “circle” with the family as illustrated in the quote above (Father, Neonatology, French interview 6), and were continuously present.

*“There were always the same persons present. One nurse in the morning, another one in the afternoon *and* during the night. So, in the end, we were with the same people for five days. That was very good.”* (Father, Neonatology, French interview 6).

ACP as a central element of PPC was mainly experienced by parents of a child with cancer, whereas families in the neurology group felt that they often had to take the lead in care planning themselves. Their engagement ensured coordination of care; however, it drained the parents of energy and strength.

The parents described their experiences concerning continuity and coordination of care in close relation to their experiences related to communication. When care was fragmented between different settings and providers such as hospital, homecare, specialists and primary carers, a gap in continuity and consequently communication was described. An especially challenging situation was described by mothers who were hospitalised in one hospital with their premature or sick newborn in another.

*“It feels like being *amputated*. Your child is gone. In another city.”* (Mother, Neonatology, German interview 5).

#### Bereavement support

For all interview parents, grieving started well before their child died and was ongoing at the time the interview took place. A variety of statements emphasising individual approaches to dealing with their loss were made; however, most parents spoke about the importance of talking about it. Parents of a newborn appreciated the support of HCPs in creating memories during the short time they had with their children.

*“We were told by the nurse to get the camera and take pictures. She said that we would never have to *look* at them if we didn’t want to, but we would have them. First, we were very shy, almost inhibited, but the nurse managed everything.”* (Mother, Neonatology, German interview 5).

For most parents, it was of utmost importance to maintain a close relationship with their child throughout EOL and dying and to be present when the child died. One mother extended the closeness after death by taking her dead child home. Another mother, due to not being informed that her child could die, because of missing information about the fact that her child could die, left the hospital and was consequently not present when her child died – an experience that still profoundly and negatively affected her at the time of the interview.

After the death of their child, the relationship with the HCPs in the hospital ended too abruptly for many parents, mainly the ones from the oncology group, and they consequently felt additional emptiness through losing emotional support from the HCPs in the hospital. Receiving cards of condolence and meeting HCPs at their child’s funeral were generally appreciated by parents.

## Discussion

This study aimed to enhance knowledge about bereaved parents’ perspectives on their child’s EOL care through connecting quantitative questionnaire survey data with qualitative interview data and contrasting positive and negative experiences. All parents in the 20 interviews described positive and negative experiences they had during their child’s illness and EOL. The deeper exploration showed that extremely negative experiences were linked to the two quality domains of communication and continuity and coordination of care. As an overarching insight, the wealth of individual experiences can also be distilled to the utmost importance of HCPs building up and being in a compassionate relationship with burdened families like these. That severely negative experiences were still haunting and bothering the parents at the time of the interview, three to four years after the loss of their child, left an unforgettable impression. A similar finding has been reported by Contro et al. [17] and confirmed by recent reviews [3, 18] and a nationwide qualitative study in the Netherlands [19]. Once again, the importance of the provision of high-quality palliative and EOL care cannot be stressed enough.

### Neonates – patients and parents with particular needs

The situation for parents losing a neonate is different in several aspects from EOL care in other patient populations [4]. For this study, the time between diagnosis and death was extremely short, with a median of four days. All neonates died in an ICU and were mechanically ventilated. Most relevantly, death was preceded by a decision to withdraw life-sustaining interventions. Experiences with shared decision-making were rated significantly lower, i.e. more negatively by parents of neonates than by parents from the other diagnostic groups in the complete sample of the quantitative survey [4]. There was a similar trend in the quantitative results of this interview sample. Experiences of good communication and shared decision-making were described positively, whereas being left alone in the decision-making process or, even worse, being pressurised by HCPs, led to negative experiences. It is recommended that shared decision-making should be achieved while recognising that the decision cannot be separated from the communication process used to reach it [20, 21] and that support should be provided through a positive and trusting relationship with the HCPs [22, 23].

Mothers who had lost their newborn children were in a unique situation because they were patients and recovering from giving birth, possibly by caesarean section. Additionally, they could have been in one hospital while the premature or sick baby was transferred to a neonatal ICU in another hospital or even another town directly after birth. Some mothers in our sample experienced insensitive and unsupportive behaviour from staff in the obstetric ward. Given this unique situation, it cannot be stated enough that staff on the obstetrics ward should treat these mothers with the utmost sensitivity and that every effort should be made to keep the mothers well informed [24, 25].

### Communication and shared decision-making – a call for compassionate care

The importance of open and honest communication and sincere and respectful relationships has been described extensively [3, 26, 27]. Extremely negative experiences related to communication were also described by some of our interviewed parents. Together with continuity and coordination of care, communication was the domain with the lowest ratings. Communication is closely linked to decision-making. Findings of what parents reported as being helpful or unhelpful related to communication or information-related characteristics in EOL decision-making have been summarised in a meta-synthesis of 58 studies [28]. Common parental complaints related to the need for more information, and to the often insensitive manner in which difficult information was delivered [19, 28]. A lack of sensitivity was experienced as especially negative by parents in our study when it was not directed to them but specifically to their child, or when their wishes on how HCPs were to communicate with their child were not respected. Direct and open disclosure of prognostic information to children has become the preferred approach nowadays. However, it should not occur in a cold, factual manner.

HCPs should adapt to individual clinical scenarios and respect the family’s wishes [29], as that is also a cornerstone of family-centred care [30]. These inter-relations may be summarised under the heading of compassionate care. However, compassion in the context of paediatric health care is as yet not well conceptualised [31]. A very recent scoping review by Sinclair et al. [31] describes factors associated with compassion in paediatric health care, particularly including continuity of care, communication and coordination. In a qualitative study aiming to define quality domains of home-based hospice and palliative care, parents created the domain of *compassionate care *[32]*.* Compassionate care was characterised by aspects of non-medical interactions promoting relationship building and contributing to perceived comfort and support [32]. Compassion can be viewed in close connection to attitudes toward family-centred and palliative care, including the awareness of the suffering of another and being in a relationship with each other as this is visualised by the father’s metaphor of the “circle” (quote of Father, Neonatology, French interview 6).

### Continuity and coordination of care – the role of advance care planning

The involvement of different healthcare providers creates challenges concerning the provision of continuous and coordinated care, a domain identified as being problematic for families of dying children. The continuity and coordination of care domain showed the lowest median score in the interview sample and the lowest mean score in the complete PELICAN sample compared to all other quality domains [4]. Many of the negative statements from parents related to relational aspects of continuity of care such as frequent changes of physicians and nurses. Continuity of care has been conceptualised in a report prepared for the Canadian Health Services Research Foundation, the Canadian Institute for Health Information, and the Advisory Committee on Health Services of the Federal/Provincial/Territorial Deputy Ministers of Health. Three types of continuity were identified: 1) Informational continuity, 2) Relational continuity, and 3) Management continuity [33]. Relational continuity refers to “the importance of knowledge of the patient as a person; an ongoing relationship between patients and providers is the undergirding that connects care over time and bridges discontinuous events.” [33]. Changing the reality of fragmented healthcare provision is difficult and necessitates particular attention within a highly-specialised healthcare system. In addition to building up a supporting and compassionate relationship with families as described above, all attempts should be made to compensate for gaps in continuity through ACP, and thorough documentation which is accessible to all parties. PPC specialists can wrap this extra layer of care around the family and other disciplines involved, designing a PC treatment plan that includes ACP and bridges gaps between providers and settings [34, 35]. Other than in PC for adults, a well-developed formalised concept for paediatric ACP interventions is lacking. Führer et al. have started to work on this important issue of care [35]. Previous initiatives have developed documents that cover particular aspects of children, such as the *Wishes Document *[36]. In this context, the role of ACP concerning PPC was evaluated [37], as was the influence of PPC on the ACP process [38].

### Bereavement support – before and beyond the death of a child

Bereavement support emerged as an area for major improvement from parental expressions in this study as in the complete PELICAN sample as well as their need for ongoing emotional support during the early period after the death of their child [4]. Families of a child with cancer commonly have close relationships with their hospital-based HCPs, since they spend a lot of time there during the (curative) oncological treatment of their child. These parents experience an additional loss of their “circle” after the loss of the child and deeply miss ongoing support from the professionals they grew accustomed to. This phenomenon emerged from a qualitative study, analysing the ongoing role of HCPs and institutions in the grief of bereaved parents of children with cancer [39] and is also reflected in a recent systematic review of parent-focused bereavement interventions [40]. The analysis of bereavement interventions by Kochen et al. [40] revealed the importance of an early start, during EOL and around the child’s death, including acknowledgement of parenthood and the child’s life, keepsakes and follow-up contacts followed by interventions at later stages of bereavement. The ongoing bond with the hospital and HCPs involved in the care of the child plays an important role in the parents’ coping and process of adjusting during grief. With respect to relationship, the changing nature of the parent-HCP partnership should be included and anticipated in the concept of compassionate care. HCPs are well aware of the changing nature of the relationship in the context of EOL care. Thus, Butler et al. [41] describe “transitional togetherness” as a core process that occurs between parents and HCPs when a child dies in the ICU. Three key phases were identified and described: 1) Welcoming expertise, 2) Becoming a team, and 3) Gradually disengaging. Parents are still in need of support when they leave the hospital after the child’s death, but this support will then be tapered off over a period of time. Butler et al. conclude that a support programme needs to be started as soon as the child dies, and ongoing contact should be offered by familiar HCPs to facilitate the process of leaving the hospital and longer-term support [41].

### Strengths and limitations

This mixed-method study was able to draw on a subsample of 30 interviewed parents from a population-based sample of parents who had lost their child due to a cardiac, neurological or oncological condition or during the neonatal period between the years 2011 and 2012. This sample might be biased in that only parents with rather favourable experiences may have decided to participate in the questionnaire survey [4]. Nevertheless, negative experiences were captured and investigated in depth through qualitative inquiry to contrast positive and negative parental experiences with their child’s EOL care. The sample size for the interviews was defined a priori, violating classical sample size determination in qualitative studies which is guided by data saturation. It is, therefore, possible that not all facets of the complex world of parental experiences were identified; however, themes recurred throughout the interviews and no new ones emerged at the end. The study’s interpretation could be transferred to the EOL experiences of parents in countries with few specialised PPC services such as Switzerland and cannot be generalised to the whole population of bereaved parents.

## Conclusions

Effective PPC requires a broad multi-professional approach, making use of all available resources in different settings and includes, as a newer concept of care, compassion for both the child and its parents [31]. A deep understanding of the perspectives and needs of parents going through the devastating event of losing a child is important and a prerequisite for providing compassionate care. This complex care needs to recognise and respond to the suffering not only of the child but the parents and the whole family. Communication and shared decision-making remain pivotal but still improvable elements of care that should build on trustful relationships between families and healthcare professionals. We should recognise and acknowledge, however, that this complex care in such a heterogeneous patient population and different care settings requires HCPs with specialised training in PPC. The development and implementation of specialised multi-professional PPC programmes are well advanced in some countries [42], whereas other countries, like Switzerland lag behind.

## Data Availability

The datasets used and/or analysed during the current study are not publicly available due to confidentiality but are available from the corresponding author on reasonable request.

## Abbreviations

ACP: Advance care planning
EOL: End-of-life
HCP: Health care professional
ICU: Intensive care unit
PaPeQu: Parental Pelican questionnaire
PELICAN: Paediatric end-of-life care needs
PPC: Paediatric palliative care
QOL: Quality of life

## References

[1] Kreicbergs UC, Lannen P, Onelov E, Wolfe J (2007). Parental grief after losing a child to cancer: impact of professional and social support on long-term outcomes. J Clin Oncol.

[2] Constantinou G, Garcia R, Cook E, Randhawa G (2019). Children’s unmet palliative care needs: a scoping review of parents’ perspectives. BMJ Support Palliat Care.

[3] Gill FJ, Hashem Z, Stegmann R, Aoun SM. The support needs of parent caregivers of children with a life-limiting illness and approaches used to meet their needs: A scoping review. Palliat Med. 2021;35(1):76-96. 10.1177/0269216320967593 33103579

[4] Zimmermann K, Bergstraesser E, Engberg S, Ramelet AS, Marfurt-Russenberger K, Von der Weid N, Grandjean C, Fahrni-Nater P, Cignacco E, Consortium P (2016). When parents face the death of their child: a nationwide cross-sectional survey of parental perspectives on their child’s end-of life care. BMC Palliat Care.

[5] Widger K, Tourangeau AE, Steele R, Streiner DL (2015). Initial development and psychometric testing of an instrument to measure the quality of children’s end-of-life care. BMC Palliat Care.

[6] Zimmermann K, Cignacco E, Eskola K, Engberg S, Ramelet AS, Von der Weid N, Bergstraesser E (2015). Development and initial validation of the Parental PELICAN Questionnaire (PaPEQu)–an instrument to assess parental experiences and needs during their child’s end-of-life care. J Adv Nurs.

[7] Widger K, Brennenstuhl S, Duc J, Tourangeau A, Rapoport A (2019). Factor structure of the Quality of Children’s Palliative Care Instrument (QCPCI) when complete by parents of children with cancer. BMC Palliat Care.

[8] Bergstraesser E, Zimmermann K, Eskola K, Luck P, Ramelet AS, Cignacco E (2015). Paediatric end-of-life care needs in Switzerland: current practices, and perspectives from parents and professionals. A study protocol. J Adv Nurs.

[9] Browning DM, Solomon MZ (2005). The initiative for pediatric palliative care: an interdisciplinary educational approach for healthcare professionals. J Pediatr Nurs.

[10] Truog RD, Meyer EC, Burns JP (2006). Toward interventions to improve end-of-life care in the pediatric intensive care unit. Crit Care Med.

[11] Creswell JW, Plano Clark VL (2011). Choosing a mixed methods design. Designing and conducting mixed methods research.

[12] Creswell JW, Plano Clark V (2011). The Nature of Mixed Methods Research. Designing and conductiong mixed methods reserach.

[13] Onwuegbuzie AJ, Bustamante RM, Nelson JA (2010). Mixed research as a tool for developing qunatitative instruments. J Mixed Methods Res.

[14] Curry L, Nunez-Smith M. Data analysis and integration in mixed methods study. In: Mixed methods in health sciences research. edn. Thousand Oaks, California, USA: SAGE Publications Inc.; 2015.

[15] Braun V, Clarke V (2006). Using thematic analysis in psychology. Qual Res Psychol.

[16] Federal Statistical Office (2015). Haushaltseinkommen und -ausgaben nach Haushaltstyp. Haushaltsbudgeterhebung (HABE).

[17] Contro N, Larson J, Scofield S, Sourkes B, Cohen H (2002). Family perspectives on the quality of pediatric palliative care. Arch Pediatr Adolesc Med.

[18] Morris S, Fletcher K, Goldstein R (2019). The grief of parents after the death of a young child. J Clin Psychol Med Settings.

[19] Brouwer M, Maeckelberghe ELM, van der Heide A, Hein I, Verhagen E (2020). Barriers in care for children with life-threatening conditions: a qualitative interview study in the Netherlands. BMJ Open.

[20] Daboval T, Shidler S (2014). Ethical framework for shared decision making in the neonatal intensive care unit: communicative ethics. Paediatr Child Health.

[21] Sieg SE, Bradshaw WT, Blake S (2019). The best interests of infants and families during palliative care at the end of life: a review of the literature. Adv Neonatal Care.

[22] Currie ER, Christian BJ, Hinds PS, Perna SJ, Robinson C, Day S, Meneses K (2016). Parent perspectives of neonatal intensive care at the end-of-Life. J Pediatr Nurs.

[23] Kharrat A, Moore GP, Beckett S, Nicholls SG, Sampson M, Daboval T (2018). Antenatal consultations at extreme prematurity: a systematic review of parent communication needs. J Pediatr.

[24] Kenner C, Press J, Ryan D (2015). Recommendations for palliative and bereavement care in the NICU: a family-centered integrative approach. J Perinatol.

[25] Parravicini E, Daho M, Foe G, Steinwurtzel R, Byrne M (2018). Parental assessment of comfort in newborns affected by life-limiting conditions treated by a standardized neonatal comfort care program. J Perinatol.

[26] Aschenbrenner AP, Winters JM, Belknap RA (2012). Integrative review: parent perspectives on care of their child at the end of life. J Pediatr Nurs.

[27] Melin-Johansson C, Axelsson I, Jonsson Grundberg M, Hallqvist F (2014). When a child dies: parents’ experiences of palliative care-an integrative literature review. J Pediatr Nurs.

[28] Xafis V, Wilkinson D, Sullivan J (2015). What information do parents need when facing end-of-life decisions for their child? A meta-synthesis of parental feedback. BMC Palliat Care.

[29] Sisk BA, Bluebond-Langner M, Wiener L, Mack J, Wolfe J (2016). Prognostic Disclosures to Children: A Historical Perspective. Pediatrics.

[30] Shields L, Zhou H, Pratt J, Taylor M, Hunter J, Pascoe E (2012). Family-centred care for hospitalised children aged 0–12 years. Cochrane Database Syst Rev.

[31] Sinclair S, Kondejewski J, Schulte F, Letourneau N, Kuhn S, Raffin-Bouchal S, Guilcher GMT, Strother D (2020). Compassion in pediatric healthcare: a scoping review. J Pediatr Nurs.

[32] Thienprayoon R, Grossoehme D, Humphrey L, Pestian T, Frimpong-Manso M, Malcolm H, Kitamura E, Jenkins R, Friebert S (2020). “There’s Just No Way to Help, and They Did.” Parents Name Compassionate Care as a New Domain of Quality in Pediatric Home-Based Hospice and Palliative Care. J Palliat Med.

[33] Reid R, Haggerty J, McKendry R (2002). Defusing the confusion: Concepts and measures of continuity of healthcare. Canadian Health Services Research Foundation, the Canadian Institute for Health Information, and the Advisory Committee on Health Services of the Federal/Provincial/Territorial Deputy Ministers of Health.

[34] Johnson LM, Snaman JM, Cupit MC, Baker JN (2014). End-of-life care for hospitalized children. Pediatr Clin North Am.

[35] Hein K, Knochel K, Zaimovic V, Reimann D, Monz A, Heitkamp N, Borasio GD, Fuhrer M (2020). Identifying key elements for paediatric advance care planning with parents, healthcare providers and stakeholders: a qualitative study. Palliat Med.

[36] Fraser J, Harris N, Berringer AJ, Prescott H, Finlay F (2010). Advanced care planning in children with life-limiting conditions - the Wishes Document. Arch Dis Child.

[37] Liberman DB, Song E, Radbill LM, Pham PK, Derrington SF (2016). Early introduction of palliative care and advanced care planning for children with complex chronic medical conditions: a pilot study. Child Care Health Dev.

[38] Harmoney K, Mobley EM, Gilbertson-White S, Brogden NK, Benson RJ (2019). Differences in advance care planning and circumstances of death for pediatric patients who do and do not receive palliative care consults: a single-center retrospective review of all pediatric deaths from 2012 to 2016. J Palliat Med.

[39] Snaman JM, Kaye EC, Torres C, Gibson DV, Baker JN (2016). Helping parents live with the hole in their heart: the role of health care providers and institutions in the bereaved parents’ grief journeys. Cancer.

[40] Kochen EM, Jenken F, Boelen PA, Deben LMA, Fahner JC, van den Hoogen A, Teunissen S, Geleijns K, Kars MC (2020). When a child dies: a systematic review of well-defined parent-focused bereavement interventions and their alignment with grief- and loss theories. BMC Palliat Care.

[41] Butler AE, Hall H, Copnell B (2018). Gradually disengaging: parent-health care provider relationships after a child’s death in the pediatric intensive care unit. J Fam Nurs.

[42] Feudtner C, Womer J, Augustin R, Remke S, Wolfe J, Friebert S, Weissman D (2013). Pediatric palliative care programs in children’s hospitals: a cross-sectional national survey. Pediatrics.

